# On the Catalytic Activity of the Engineered Coiled-Coil Heptamer Mimicking the Hydrolase Enzymes: Insights from a Computational Study

**DOI:** 10.3390/ijms21124551

**Published:** 2020-06-26

**Authors:** Mario Prejanò, Isabella Romeo, Nino Russo, Tiziana Marino

**Affiliations:** Department of Chemistry and Chemical Technologies, University of Calabria, 87036 Arcavacata di Rende, Cosenza, Italy; mario.prejano@unical.it (M.P.); isabella.romeo@unical.it (I.R.)

**Keywords:** Free-energy surface (FES), rate-determining step, density functional theory, molecular dynamics, hydrolase, de novo protein, mechanism

## Abstract

Recently major advances were gained on the designed proteins aimed to generate biomolecular mimics of proteases. Although such enzyme-like catalysts must still suffer refinements for improving the catalytic activity, at the moment, they represent a good example of artificial enzymes to be tested in different fields. Herein, a de novo designed homo-heptameric peptide assembly (CC-Hept) where the esterase activity towards p-nitro-phenylacetate was obtained for introduction of the catalytic triad (Cys-His-Glu) into the hydrophobic matrix, is the object of the present combined molecular dynamics and quantum mechanics/molecular mechanics investigation. Constant pH Molecular Dynamics simulations on the apoform of CC-Hept suggested that the Cys residues are present in the protonated form. Molecular dynamics (MD) simulations of the enzyme–substrate complex evidenced the attitude of the enzyme-like system to retain water molecules, necessary in the hydrolytic reaction, in correspondence of the active site, represented by the Cys-His-Glu triad on each of the seven chains, without significant structural perturbations. A detailed reaction mechanism of esterase activity of CC-Hept-Cys-His-Glu was investigated on the basis of the quantum mechanics/molecular mechanics calculations employing a large quantum mechanical (QM) region of the active site. The proposed mechanism is consistent with available esterases kinetics and structural data. The roles of the active site residues were also evaluated. The deacylation phase emerged as the rate-determining step, in agreement with esterase activity of other natural proteases.

## 1. Introduction

Proteases are a family of enzymes whose function is to catalyze the hydrolysis of peptide bonds. Although physiologically these enzymes are peptide bond hydrolases, they can also promote the hydrolysis of substrates of different natures such as esters, thioesters, anhydrides, and acid chlorides.

Despite the differences in structure, active site, and metal center present in the four main classes of proteases (serine proteases, cysteine proteases, aspartyl proteases, and metalloproteases), all of them have in common the use of a nucleophile whose activation can occur in different ways. In particular, serine, and cysteine proteases use a catalytic triad to activate the side chain of a serine and cysteine, respectively.

Previous studies in mechanistic enzymology, choosing the chymotrypsin as a prototype for a variety of enzyme classes including proteases, allowed to evidence the peptide hydrolysis occurring in different steps (activation, acylation, and de-acylation). The activated nucleophile attacks the carbon of the peptide bond and the electrons in the carbon-oxygen double bond migrate onto the oxygen. This formed tetrahedral intermediate is a high energy species and the enzyme will generally take care to stabilize it through an oxyanion hole that is a part of the protease. 

It is well known that the peptide bond hydrolysis of a peptide bond is not an easy reaction since the kinetic constant of uncatalyzed reaction (k_uncat_) value measured at pH 9 and 25 °C is 8.2 × 10^−11^ s^−1^, in analogy with the conditions of the enzymatic reaction, suggests a very long half-life (about 168 years) for hydrolysis in the absence of enzyme [1]. For this reason, the knowledge of the reaction mechanism is very important not only to understand the phenomenon but also to suggest solutions for new catalytic systems.

Recently, the coiled-coil heptamer (CC-Hept), the outcome of a rational computational design of a series of α-helical barrels [2,3,4] has been used for the installation of functional residues “with precision and within defined environments” suited to mimic active site present in very common natural cysteine/serine hydrolases family [5].

In particular, the CC-Hept having three mutations per chain has been designed for mutation of hydrophobic residues (leucine and isoleucine) with cysteine, histidine, and glutamic acid (Cys-His-Glu), projecting into the central pore. This de novo protein is a good example of the engineered enzyme where short sequences of amino acids (three in this case) are used to reproduce the active site of hydrolase enzymes. Although the activity of this construct is much lower than that of a native enzyme [2], a rudimentary catalytic activity in ester hydrolysis has been observed. Such a low catalytic efficiency also minor than a recent in silico designed metalloprotein [6], calls for the improvement both efficiency and selectivity of the CC-Hept. 

**Scheme 1:**
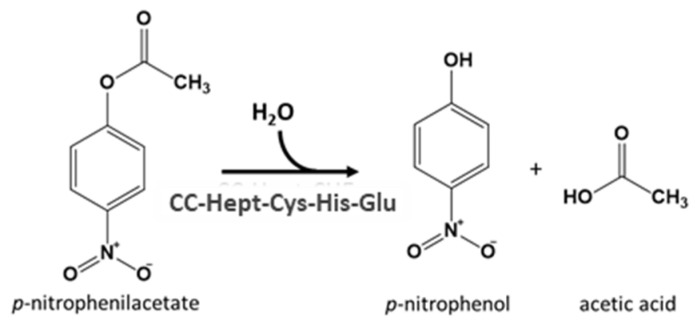
Hydrolysis of p-nitrophenyl acetate (pNPA) promoted by CC-Hept-Cys-His-Glu.

To the best of our knowledge, insights on the mechanism for hydrolytic activity into de novo protein, unlike the natural Cys/Ser hydrolases [7], are not yet explained in the literature. Motivated from the growing interest towards these de novo protein scaffold catalytic machineries, we have undertaken a study based on both molecular dynamics simulations (MDs) and the hybrid quantum mechanics/molecular mechanics (QM/MM) calculations, to gain knowledge on the mechanism (see Scheme 1) and to rationalize, on the basis of the quantified free energy surface, the not so very amazing observed catalytic activity of the CC-Hept containing the triad, Cys-His-Glu. 

We used the p-nitrophenyl acetate (pNPA), as a substrate, in agreement with the experimental study [2]. Since the study of enzymatic activity of hydrolases gave important information on the catalytic mechanism of this class of enzymes [1]. Additional information on the esterase activity of CC-Hept-Cys-His-Glu arising from the present mechanistic investigation may be helpful for enzyme design of enhanced versions of hydrolases to be applied in biotechnology and biochemical synthesis. 

## 2. Results

### 2.1. MD Investigation

**Figure 1:**
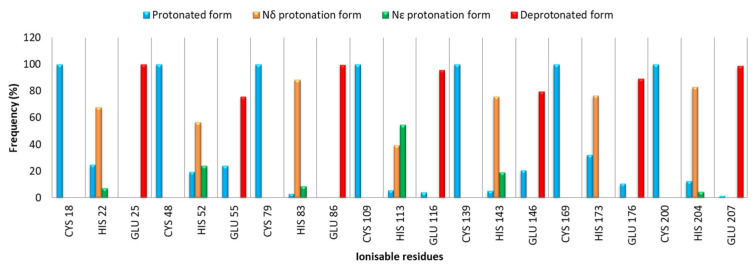
Populations of every protonation state in terms of frequency (%) for 21 ionizable residues.

In the first step of our investigation, we have determined the protonation state of the catalytic triad residues for all the seven chains, starting from the X-ray model of the heptameric coiled coil apo-form, (PDB code 5EZC, see Figure S1) [2]. Both static and dynamic applied methods suggested that all the Cys and Glu residues are in the protonated and deprotonated form, respectively (Table S1). In the case of histidine residue, the Nδ site resulted to be the most preferred one even if from Constant pH Molecular Dynamics (CpHMD) (Figure 1) emerged also a configuration with six His protonated in Nδ and one in Nε. This behavior leads us to perform MDs of 200 ns for all the three systems as it follows: one with all the His protonated in Nδ, the other with all the His protonated in Nε and the other again with six His protonated in Nδ and the remaining one protonated on Nε.

**Figure 2:**
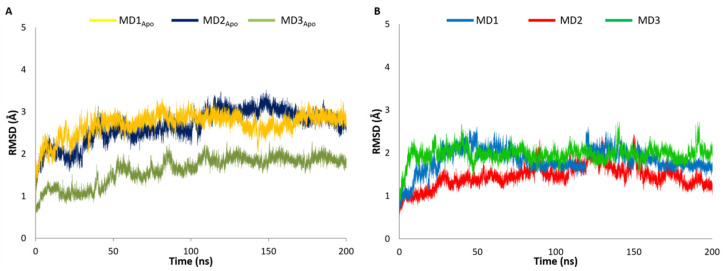
*(***A**) Root Mean Square deviations RMSD (in Å) with respect to the time in nanoseconds for the CC-Hept-Cys-His-Glu apo-form. The simulations with all the His Nε, Nδ and the structure with six His are in Nδ and one in Nε, correspond to yellow, blue and green lines, respectively. (**B**) Root Mean Square deviations RMSD (Å) with respect to the time in nanoseconds for the three different conformations of pNPA/CC-Hept-Cys-His-Glu.

The RMSD plot of the analyzed trajectories for the three apo-form structures of CC-Hept-Cys-His-Glu depicted in Figure 2A clearly shows that the system characterized by the RMSD average value of ~1.60 Å (green line) corresponding to all His protonated on Nδ site is the most stable one. This finding well agrees with the X-ray structure [2] and induced to consider the MD2_Apo_ as a starting point for the subsequent investigations. In fact, the superposition of the ten selected representative structures from clustering analysis performed on the 200 ns of the MD2_Apo_ MDs (Figure S2) well highlighted the conformational homogeneity of the chains in particular in correspondence of the bottom region of CC-Hept-Cys-His-Glu (1–15 residues). 

By molecular recognition of pNPA [2,8] into the channel of the de novo protein, ten complexes were generated for each obtained representative structure (Figure S3). Based on the applied geometrical filters aimed to ensure the nucleophilic attack of the Cys thiol on the pNPA and on the best binding affinity value, (see Table S2) three conformations were chosen for further 3 × 200 ns of MDs with the aim to monitor the pNPA behavior in correspondence of the active site. 

By MDs, we observed that the overall protein structure remained stable during the simulation time, as confirmed by the average RMSD values (1.10 Å, 1.60 Å, and 2.01 Å) of CC-Hept-Cys-His-Glu in complex with the substrate (Figure 2B). The RMSDs trend revealed that, in the presence of pNPA, the structures were associated with the higher stability with respect to the unbound forms, underlying a slight tightening in response to the interaction with the substrate as previously observed in other de novo designed protein [9].

The analysis of the pair correlation function shown in Figure S4, demonstrates the presence of a single peak related to the closest contact between the thiol group of the seven cysteines and the carbonyl of the substrate at about 6 Å, in all the three MDs.

**Figure 3:**
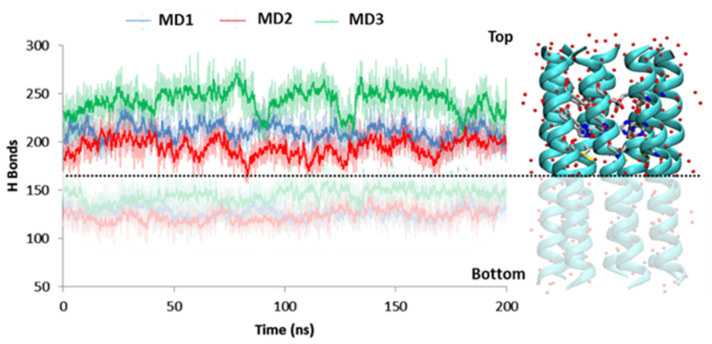
Hydrogen bonds number (H-bonds) between water molecules and donor/acceptor atoms of side chains inside the channel as function of the time (ns) during the whole trajectories of MD1, MD2, and MD3. The dashed black line separates the number of H-bonds calculated in the top (16–30 residues) and in the bottom region (1–15 residues) of CC-Hept-Cys-His-Glu.

Due to the diameter of the channel of CC-Hept-Cys-His-Glu, as it can be evinced from the trend of the diameter in top, middle and bottom shown in Figure S5, the substrate is able to move and rotate freely in proximity of the active site described by the Cys-His-Glu catalytic triad. In presence of pNPA, the protein showed a slight tightening around catalytic triad, likely due to the increased number of interactions between protein and substrate into the channel promoted also by water molecules close to the active site, that must come into play in the hydrolysis of thioester intermediate. As it can be evinced by Figure 3, the number of water molecules is higher in proximity to the catalytic triad close to the top of the channel as confirmed by the H bond interactions that become almost twice as much as those close to the bottom. This behavior well accounts for the rationale to obtain a region more accessible to water molecules upon the installation of more hydrophilic residues in a hydrophobic scaffold [2].

### 2.2. Reaction Mechanism 

**Figure 4:**
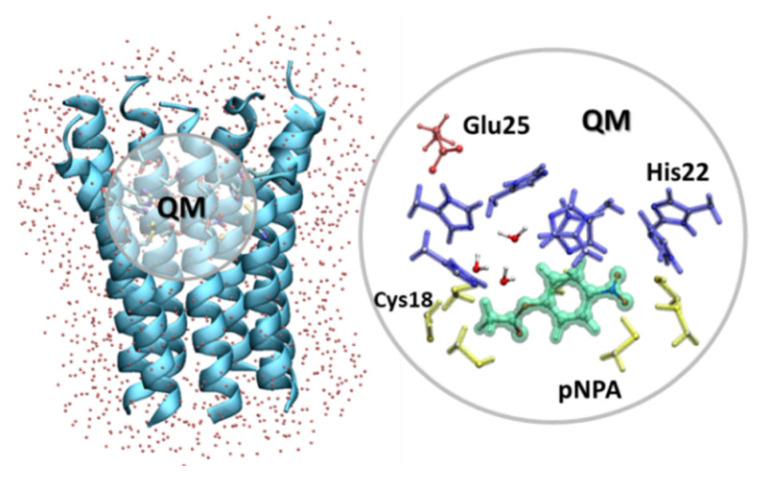
The adopted model for QM/MM investigation. Water molecules within 5 Å around the protein are shown as red dots; Cys, His and Glu residues are distinguished with colors of yellow, blue, and red ball and stick, respectively. The pNPA substrate is in green.

In order to investigate the catalytic mechanism, hybrid QM/MM procedure was applied as previously reported in similar systems [10,11,12,13,14,15,16]. The QM region includes Cys18, His22 of the seven chains and Glu25 faced into the hydrophobic channel of the CC-Hept-Cys-His-Glu and three water molecules (w1, w2 and w3) engaged in a hydrogen bonds network (see Figure 4) both with each other and with the catalytic triad (Figure S6). Detailed description of the model’s selection is reported in the Methods section. 

The hydrolysis reaction requires a real consumption of a water molecule, so the choice to retain three water molecules in the QM region can provide an adequate network of H-bond interactions present in proximity of the hydrophilic active site (Cys-His-Glu) as also observed during the MDs. As above mentioned, the hydrolysis reaction occurs in two phases following a ping-pong Bi-Bi mechanism: the “initial burst”, the fast initial phase in which the enzyme very quickly reacts with the pNPA releasing the highly colored p-nitrophenolate (the first product, **P1**) and forming the acyl-enzyme covalent intermediate (enzyme-second substrate complex, acylation step) that in the second one, the linear steady-state phase (deacylation step), is slowly hydrolyzed in free enzyme and acetate (the second product, **P2**). Really, the ester hydrolysis follows a reaction process very similar to the amide hydrolysis but differs in the rate-determining step that is the deacylation phase.

**Scheme 2:**
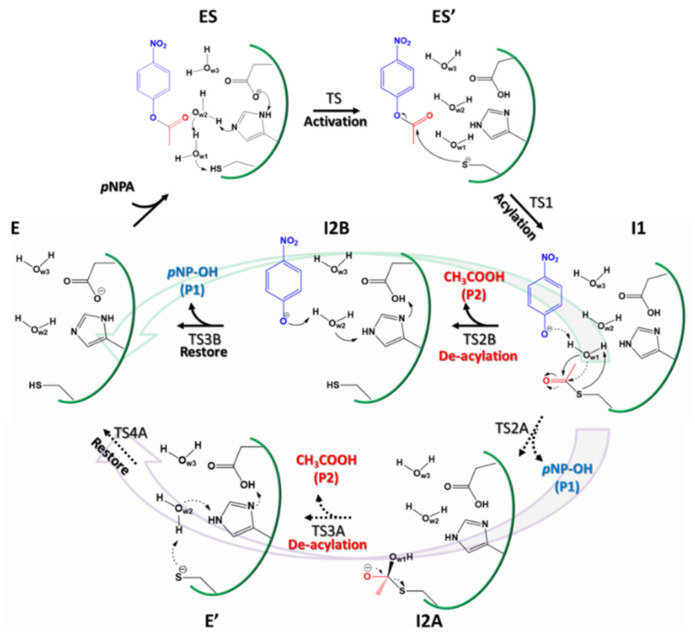
Two proposed reaction mechanisms of pNPA hydrolysis catalyzed by de novo protein. From I1 the reaction can proceed in two elementary steps for the channel A (continuous lines) and three elementary steps for the channel B (dotted lines).

The details of our proposed mechanism are given in Scheme 2. 

In the reported mechanism is included also the conversion step, ES→ES′, corresponding to the deprotonation of Cys residue by w1 that acts as the proton shuttle to His linked by hydrogen bond to the H^+^ final acceptor Glu. 

In the acylation process, the newly formed active nucleophile Cys-S^(−)^ can perform the nucleophilic attack on the electrophilic carbon of the reactive ester (pNPA) with the synchronous leaving of p-nitrophenolate anion (**P1**) and the formation of a thioester (**TS1**). From the resulting species (**I1**) the deacylation process can occur through two different channels (A or B) for affording the hydrolysis products. In the channel A, three elementary steps are required for obtaining the CC-Hept-Cys-His-Glu restore. In the first one (occurring via **TS2A**) w1 performs a nucleophilic attack on the electrophile carbon of the thioester generating a tetrahedral intermediate (**I2A**). Subsequently, the CH_3_COOH formation with the consequent C-S bond cleavage (**TS3A**) occurs giving rise to **I3A**. Finally, the restoration of the initial protonation state of the Cys-His-Glu triad is promoted by w2. 

In the channel B, w1 comes into play by nucleophilic attack on the carbon of the thioester and simultaneously transfers the proton to sulfur atom generating the C-S bond cleavage (**TS2B**) with the acetic acid release and –SH moiety restoration on the Cys (**I2B**). In the last step, His accomplishes the process acting as a general acid/base to deprotonate the Glu residue for protonating w2 that in turn gives delivers the proton to p-nitrophenolate ion (**TS3B**) regenerating the catalytic triad.

**Figure 5:**
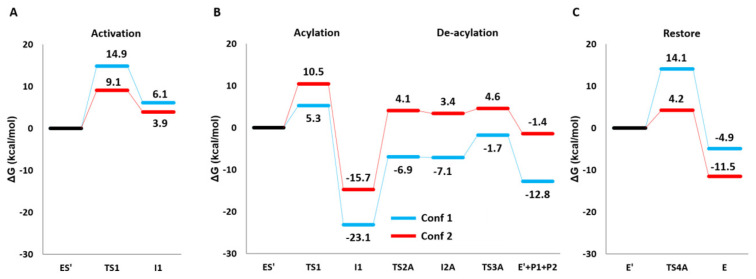
B3LYP-D3/6-311+(2d,2p):ff14SB//B3LYP/6-31+G(d,p):ff14SB Free Energy Surfaces for pNPA hydrolysis catalyzed by de novo protein, according to the A mechanism for the considered conformations (aligned triad-Conf 1-blue line and unaligned triad-Conf 2- red line). (Panel **A**) activation step; (Panel **B**) Acylation and De-acylation step; (Panel **C**)Restore step.

Only the computed free energy surfaces (FESs) related to the channel A (Figure 5) will be discussed, while those of the channel B are given in supporting information since the channel A well depicts the release of **P1** followed by that **P2** in agreement with the experimental counterpart [2] and with the typical ping-pong mechanism well established for α-chymotrypsin with the unnatural substrate pNPA [17,18]. In particular, two starting structures named conformation 1 and conformation 2, characterized by the perfectly aligned triad as present in X-ray and the other one by the unaligned triad, respectively were used in the QM/MM mechanistic investigation.

**Figure 6:**
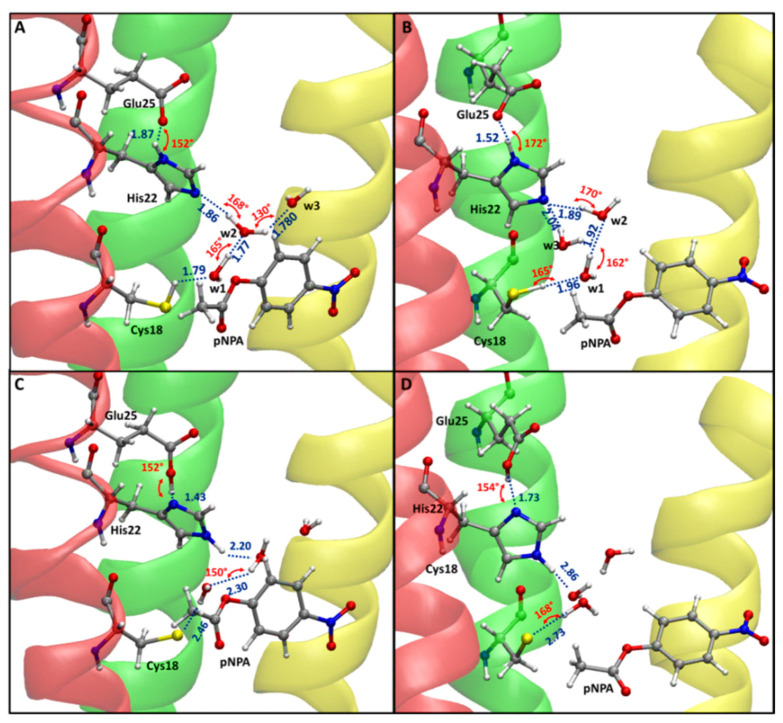
B3LYP/6-31+G(d,p):ff14SB optimized geometry optimized geometry of Cys is in neutral form (ES) (**A**,**B** of conformation 1 and 2, respectively) and ES′ (**C**,**D** of conformation 1 and 2), respectively In details, in blue and red label, the distance and the angle are shown, respectively. Main distances (Å) between water molecules and residues are reported in black dots. For clarity, only the amino acid (Cys18-His22-Glu25) residues taking part in the reaction are shown and along with pNPA are represented in ball and sticks.

On the basis of our results of the preliminary static and dynamic evaluation of the protonation states of the triad, we optimized the geometry of the enzyme–substrate complex with Cys in the neutral form, shown in Figure 6. Furthermore, in order to test which form is really responsible for the nucleophilic attack, we have considered the thiol and thiolate in the nucleophilic attack on the acyl group of pNPA. Our calculations showed that the neutral thiol group is unable to perform the nucleophilic attack since the energy increases throughout the entire linear transit scan (above 40 kcal/mol) (Figure S7), while the deprotonated Cys appears as the reactive form of the nucleophile attack.

From this evidence, it resulted mandatory the activation of the thiol group (from the neutral to the deprotonated state) and the related FES is depicted in Figure 5 Panel A. Our QM/MM calculations propose that the Cys residues of the CC-Hept-Cys-His-Glu in the neutral form represent the resting state of the artificial enzyme. In fact, the energy of the enzyme–substrate complex in which Cys is in neutral form (ES) is more than 3 kcal/mol (6 kcal/mol) lower than when Cys is deprotonated (ES′) for unaligned (aligned) conformation. (see Figure 5 Panel A) In addition, this finding is consistent with the fact that at experimental pH (7.0) [2] the Cys characterized by the intrinsic pKa ~8.6 [19] is protonated. 

The deprotonation of the cysteine in active site of cysteine proteases represents a crucial step for catalysis performed for these hydrolases [20,21]. In de novo protein, the thiol deprotonation is promoted by w1 and w2 wrapped between His and Cys residues, that facilitate the delivery of proton to the general base Glu residue and this step required an activation energy (TS) equal to 9.1 and 14.1 kcal/mol for unaligned and aligned triad, respectively (Figure 5 Panel B). Natural charges, arising from the NBO analysis confirm the increased nucleophile character of the soft sulphur atom in the deprotonated form related to the neutral form (Table S5). The TS vibrational modes, see Table S6, of about 892*i* cm^−1^, for the two conformations display a linear stretching of the three O_w1_—H—O_w2_ atoms. In all cases, w1 and w2 promote the concerted proton shifts starting from the thiol of Cys until the oxygen of Glu (Figure S8). These movements induce a rotation of pNPA in the acetyl terminal region that assumes a trans geometry more suitable for the next nucleophile attack. From this behavior appears the first difference with other cysteine proteases where the Cys and His residues are directly involved in the proton transfer to generate the highly nucleophilic Cys-S- ion without water assistance [22,23,24,25] The optimized geometries of **ES** of conformation 1 and 2, are shown in Figure 6A,B, respectively.

The two **ES′** species containing the activated Cys-S^(−)^ are shown in Figure 6C,D. 

From **ES′** the nucleophilic attack of Cys on the carbonyl carbon takes place to form a covalent bond between the enzyme and the substrate. (acylation) 

The **TS1** (10.5 and 5.3 kcal/mol for conformation 1 and 2, respectively above the **ES′**) clearly depicts the nucleophilic attack by Cys-S^(−)^ on the C (e.g., 2.23 Å in conformation 2) with the consequent elongation of the C-O bond and release of the p-nitrophenolate anion (Figure S9). The related imaginary frequency of 150*i* cm^−1^ well accounts for the S-C and C-O stretching movements. This barrier is in agreement with the measured k_2_ value of 83 s^−1^ [2], related to the rate constant of the acylation process which according to classical transition state theory, can be converted to 15 kcal/mol. The rate of the acylation step in the ester hydrolysis follows an opposite behavior to that of the amide hydrolysis where as soon as the acyl intermediate forms, it quickly undergoes deacylation [17]. 

The formed **I1** intermediate (thioester) proposes the acyl intermediate bound to the Cys and is much more stabilized with respect to **ES′** (see Figure 5 Panel B). 

**Figure 7:**
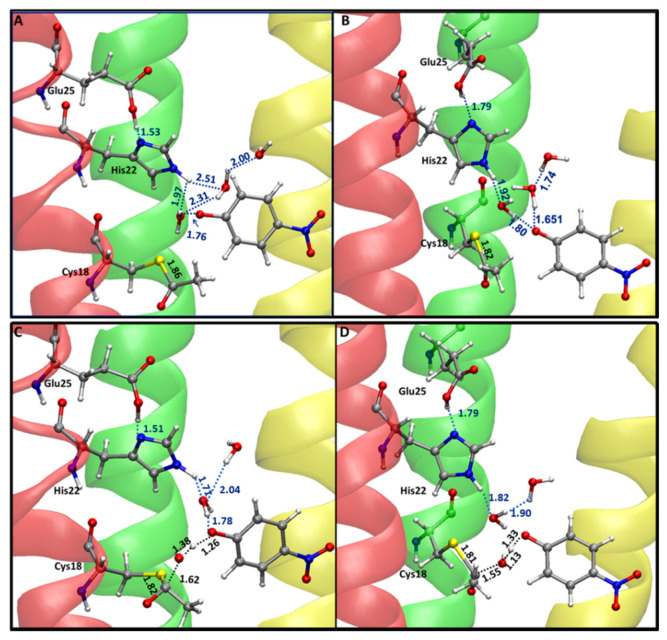
B3LYP/6-31+G(d,p):ff14SB optimized geometry optimized geometry of I1 (**A**,**B** of conformation 1 and 2, respectively) and TS2A (**C**,**D** of conformation 1 and 2), respectively and respective hydrogen bond network are reported. In detail, in blue, the distances are shown. Main distances (Å) between water molecules and residues are reported in black dots. For clarity, only the amino acid (Cys18-His22-Glu25) residues taking part in the reaction are shown and along with pNPA are represented in ball and sticks.

As previously mentioned, the reaction from **I1** species can follow two different channels (see Scheme 2). In the channel A, the reaction proceeds throughout **TS2A** that describes the protonation of pNPA occurring in a concerted way with the formation of tetrahedral intermediate promoted by w1 (Figure 7). 

The calculated barrier for this step is 16.2 kcal/mol relative to **I1** for conformation 1 (19.8 kcal/mol for conformation 2) (see Figure 5 Panel B). This step of deacylation represents the rate-limiting step of the reaction and well compares with the experimentally measured k_cat_ of 5 s^−1^ [2] that corresponds to a barrier of ca 22 kcal/mol. The formed **I2A** species is a very short-lived intermediate and reflects the above-explained rearrangement with a more lengthened C—S bond (2.02 Å in the aligned triad and 2.20 Å in the unaligned one, Figure S10). 

From **I2A** a barrier (**TS3A**) by about 5.4 kcal/mol for conformation 1 (1.2 kcal/mol for conformation 2) must be overcome to obtain **E′ + P2**. This species is characterized by the presence of the deprotonated form of Cys that in turn must undergo a protonation to generate the enzyme-like CC-Hept-Cys-His-Glu ready to restore the catalytic cycle. To do this, multiple proton shifts between the thiolate group of Cys and imidazole ring of His, promoted by w2 water molecule, take place with a modest energetic expense as described by the **E′-TS4A-E** step. (see Figure 5 Panel C). This last step corresponds to the reverse of the activation step (Figure 5 Panel A) but the found energetical differences (by about 5 kcal/mol for both considered forms) between them well account for the loss of H-bond interactions due to the consumption of a water molecule. As occurred in the activation, the stationary point presenting the neutral thiol (**E′**) presented lower energy respect to that containing the thiolate (**E**), in both conformations. 

## 3. Discussion

Our preliminary structural analysis underlined the stability of CC-Hept-Cys-His-Glu assembly, thus promoting multimeric association, and installing peculiar active-site residues with hydrolytic activity due to the polar/non-polar sequence periodicity of the α-helical barrel [2].

MDs revealed that the presence of the pNPA led to a top channel growth to accommodate it, if compared to the apo-form protein simulation. Due to the diameter of the channel of CC-Hept-Cys-His-Glu, the substrate is able to move and rotate freely in proximity of the binding site comprising Cys-His-Glu catalytic triad. In presence of pNPA, the protein showed a slight tightening around catalytic triad, likely due to the increased number of interactions between protein and substrate into the channel promoted also by water molecules present in the catalytic pocket, thus taking part in the crucial steps of the reaction mechanism.

The QM/MM free energy surfaces obtained by using two different initial representative structures for the **ES** show that in the common first part of the reaction mechanism (activation) the energy of conformation 1 is higher than conformation 2. This can be explained considering that in the **ES** of this structure the orientation of the S-H group is different and the distance between the sulfur atom and the Nε of His results to be longer (5.30 Å) and not ideal for an efficient H-transfer.

Furthermore, we note as the topology of the CC-Hept-Cys-His-Glu does not allow the formation of the oxyanion hole in the acylation step of the reaction (**ES′**-**TS1**-**I1**), observed in other esterases [5,26], since the only backbone N-H groups are engaged in the formation of α-helical barrels and so, are not willing to provide the partial ionic charge at the active site useful to stabilize the potential opposite charge developing on the substrate at this step of the reaction. The barriers for the acylation resulted to be 5.3 kcal/mol and 10.5 kcal/mol, from the **ES′** species for conformation 1 and 2, respectively. 

This aspect for the present engineered catalyst represents an additional corroboration that the nucleophilic attack occurs simultaneously with the release of the most stable p-nitrophenolate species without the tetrahedral intermediate.

The calculated thioester stabilization (**I1**) is in very good agreement with its experimental isolation and characterization by mass spectrometry [2].

From our FESs it is clear that the first step of the reaction (activation of the cysteine and the activation requires a minor energy expense. This results to be a peculiarity of this artificial protease, since in the natural one generally, the nucleophilic attack is the rate-limiting steps [27].

For the deacylation process, two different pathways have been taken into account, starting from **I1** intermediate. In the channel B, the water attack to the carbonyl of thioester to restore the S-H(Cys) group with the concomitant release of the first reaction product (acetic acid) requires the overcoming of energetic barriers of 32.9 kcal/mol and 22.5 kcal/mol, for conformation 1 and 2 respectively. On the contrary, in the channel A, where the hydrolysis occurs through the formation of the tetrahedral intermediate (**I2A**), the energy demand is equal to 19.8 kcal/mol and 16.2 kcal/mol, for conformation 1 and 2, respectively. This indicates the channel A as favored one and **TS2A** represents the rate-limiting step in agreement with what proposed by the experimental counterpart [2]. 

In any case, we underline that the used substrate pNPA present different properties with respect to the usual substrates of natural proteases, since its leaving group is more reactive. Furthermore, we note as the topology of the CC-Hept-Cys-His-Glu does not allow the formation of the oxyanion hole, noted in other esterases [28,29], due to the engagement of the backbone N-H in the formation of α-helical barrels. 

With this in mind, our results are useful for rationalizing the experimental findings on this system [2] and also can be able to drive future improvement of these de novo assemblies. 

## 4. Materials and Methods 

### 4.1. MDs for CC-Hept-Cys-His-Glu and pNPA/CC-Hept-Cys-His-Glu 

Starting from the X-ray model of the heptameric coiled coil apo-form, (PDB code 5EZC) [2], MDs were performed by using AMBER16 [30] package (Figure S1). The initial configuration was refined by using xLEaP [31] and Amber ff14SB [32] as a force field. The protonation states of the ionizable Cys, His and Glu residues were evaluated by means of both the static H++ method [33] and the dynamic one based on explicit-solvent constant-pH molecular dynamics (CpHMD) for a total of 21 ionizable residues [34]. Results arising from H++ method are collected in Table S1. Protonation states statistics were computed with AMBER16 employing the *cphstats* tool (San Francisco, CA, USA) [30]. 

Subsequently, with the aim to analyze the influence of the protonation states of the histidine, three different MDs of 200 ns were done, taking into account three different combinations of the histidine protonation states (Nδ, Nε, Nδ/Nε). The system was solvated in an orthorhombic box with a buffer of 10 Å, using TIP3P water molecules, meanwhile, the counter ions (Cl^−^) were added within 2 Å of the protein in order to neutralize the net charge. 

The solvated structure was first minimized by applying positional harmonic restraints on all atoms (50 kcal/mol Å^2^) using 5000 steps of steepest descent [35] followed by 5000 steps of conjugate gradient. In the second minimization step, the whole system was released without any restraint and then a progressive heating phase was carried out from 0 to 300 K for 50 ps, followed by 50 ps at 300 K using the Langevin thermostat in NVT ensemble. The production phase was performed for 200 ns of MDs under the following conditions: integration step of 2 fs coupling SHAKE algorithm, NPT ensemble at 1 bar pressure using the Berendsen barostat [36] with a time constant τp = 2.0 ps. The Particle mesh Ewald summation method [37] was employed for the electrostatic potential and the long-range electrostatic interactions were calculated with 12 Å cut-off distance. In order to select different representative conformations of the system, we performed RMSD-based clustering of the whole trajectory according to the relaxed complex scheme docking protocol [8,38]. After removing overall rotations and translations by RMS-fitting the Cα atoms positions of the trajectory, we applied the average linkage clustering algorithm, implemented in *cpptraj*, identifying 10 representative conformations for each simulation. 

These structural insights allowed both the local and global flexibility of the protein and decreased the computational cost for the relaxed complex scheme docking procedure [39]. Afterwards, 10 clusters characterized by the His-Nδ protonation were considered for the molecular docking of the pNPA into CC-Hept-Cys-His-Glu system. For each starting point, the AutoDock Vina [40] program generated 10 output poses (Figure S3). Box centroid was determined by a geometric center of the seven Cys residues involved into the substrate-binding region and a box of 32 Å size for X, Y, and Z was used for grid point generation. From the obtained 100 binding poses, the 10 lowest energy ones were selected. Furthermore, imposing the geometrical criteria (distance cut-off equal to 6 Å for S_Cys_-C_pNPA_, S_Cys_-Nε_His_, and Nδ_His_-O_Glu_) structures were extracted for 200 ns of MDs each one (Table S2). The parameters for the pNPA substrate molecule were calculated from single molecule optimization at HF/6-31G(d) level of theory. The general amber force field (GAFF) [41] and the restrained electrostatic potential (RESP) [42] methods were employed to obtain intramolecular and Lennard-Jones parameters and atomic charges, respectively (Table S3). 

After cluster analysis of the obtained MD trajectories and by using the previous energetic and geometrical criteria (Table S4), we selected two structures (one containing the perfectly aligned triad (conformation 1) as present in X-ray and the other an unaligned triad (conformation 2)) as starting points for the next QM/MM calculations. Both conformations ensure a good orientation for catalysis since all the Cys-His-Glu triads are included within hydrogen bonding distance between them. Furthermore, this choice gives the opportunity to study the influence of the triad disposition on the catalysis behavior. 

### 4.2. QM/MM 

In the QM/MM structures, the water molecules within 5 Å from protein were retained and a two layers ONIOM formalism was used [43]. The QM portion consists of 141 atoms (*p*NPA, Cys18_A-G_, His22_A-G_, Glu25_A/G_ along with three water molecules close to active site, see Figure 4) and has a total charge equal to −1. The remaining atoms of the system are included in the MM region. 

During the optimizations, water molecules around the protein were kept frozen in their positions. ONIOM calculations were carried out using Gaussian 09 [44] software package, applying the electrostatic embedding scheme [45]. The link atom approach as implemented in Gaussian 09 consisting in saturating the valences on the atoms where the truncation of bonds across the DFT and MM layers has been applied. The B3LYP/6-31+G(d,p) [46,47] and ff14SB [32] levels of theory were used for all geometry optimizations, for QM and MM regions respectively. Different previous works supported the use of B3LYP functional in enzymatic catalysis [10,11,12,13,48,49]. Full geometry optimizations and frequencies calculation for all the stationary points of the free energy surfaces were performed. In particular, the presence of a single imaginary vibrational frequency confirmed the nature of maxima (transition states) at the contrary non-imaginary frequencies the nature of minima.

In order to quantify entropic contributions, the Grimme’s procedure [50] was employed as reported in other works [14,15,16]. According to this method, only vibrational temperatures higher than 100 K are taken into account to calculate *TΔS* contribution using the harmonic oscillator/rigid rotor/particle in a box formalism. The final free energy surfaces were obtained including electronic energies, calculated with the larger 6-311+G(2d,2p) basis set, the zero point energy (ZPE) corrections, entropy contributions and empirical-D3 dispersion corrections [51], as reported in other works [14,15,16,49]. The natural bond orbitals analysis (NBO) [52] as implemented in Gaussian 09 code was carried out on each stationary point intercepted along the free energy surfaces.

## 5. Conclusions

In the present work, we have undertaken a detailed study based on both MD simulations and QM/MM calculations to investigate the ester hydrolysis promoted by a totally engineered protein converted into hydrolase enzyme for installation of the common Cys-His-Glu triad in cysteine proteases, on every chain of the coiled-coil heptamer. The outcomes from the used computational protocol well fit the hypotheses from the available experimental data revealing to be adequate to describe the conformational behavior of this enzyme-like catalyst in both apo- and bound- forms. MDs evidenced that the water molecules are mainly distributed on the top of the channel where the Cys-His-Glu installation occurred. The QM/MM structures were obtained by MDs that gave back two low lying energy conformations characterized by a different disposition of the triad and a detailed energy profile was produced for the complete catalytic cycle including the activation, hydrolysis reaction and restore steps. The resulting FESs helps to better rationalize the experimental observations. The calculations show that the nucleophilic attack by Cys on the carbonyl carbon of the substrate takes place after the thiol activation. This proton transfer is facilitated by His residue that acts as a general base with the assistance of two water molecules. An important prediction of the calculations in fact is that the neutral Cys is present in the resting state of the enzyme-like catalyst, CC-Hept-Cys-His-Glu. The acylation step corresponding to the burst phase occurs very quickly. Our investigation demonstrates that the formation of the tetrahedral intermediate following the nucleophile attack did not take place, confirming that the lacking of oxyanion hole is a fundamental requirement for the peptide/esters hydrolysis. The reaction proceeds via thioester intermediate formation in agreement with the experimental findings as confirmed by the lowest energy species (**I1**) independently of the considered catalytic triad alignment. The two considered conformations for the mechanistic investigation remark that the triad disposition is not discriminant for the purposes of catalysis since both generate free energy surfaces with comparable energetics. This behavior could be the consequence of the peculiar assembly of the protein that collects all the linear triads so they can contribute to influence together the catalytic activity, even if this is not the sole requirement for having a more performant efficiency. The rate-determining step (channel A) of the de-acylation step lying at 19.8 and 16.2 kcal/mol with respect to the **I1** species, for the conformation 1 and 2, respectively demonstrates that the triad alignment as observed in the X-ray structure generates the barrier 3.6 kcal/mol lower than that in the aligned one. The calculated barrier is in good agreement with the experimental *k_cat_* value, taking into account the B3LYP overestimation (1–3 kcal/mol). The nature of the de-acylation step as rate-limiting step is in agreement with other esterase available enzymes. The restore step takes place with energetics very similar to that of activation. The present calculations provide good insights into the installation of the hydrophilic triad into hydrophobic confined space helpful to assist the future rational design of other more efficient engineered functionalized enzyme-like catalysts.

## Supplementary Materials

The following are available online at https://www.mdpi.com/1422-0067/21/12/4551/s1, Figure S1. The initial structure adopted in the present study (PDB: 5ECZ), Figure S2. Superposition between 10 representative structures generated by cpptraj, after 200 ns of the apo-form protein MDs with Nδ protonation state in (A) top and (B) front view. Each structure is represented in ribbon form, Figure S3. Representation of the unnatural substrate pNPA binding mode into the channel of the de novo protein for each obtained representative structure. 10 poses are generated by using AutoDock Vina. The protein and the relative pNPA binding poses are shown in light grey cartoon and coloured carbon sticks, respectively, Figure S4. The pair correlation function for the sulphur of cysteine and the carbon of pNPA, Figure S5. Plot of the distance calculated between the centroids of all the seven Gly1, Cys18 and Arg30 residues of the protein and the corresponding Cα of Gly, Cys and Arg of each chain as a measure of the diameter of the channel at bottom (blue line), middle and top (green and red lines), respectively, relative to the initial structure. The time of simulations (ns) and the values of the Cα distance (Å) are reported, respectively, on the abscissa and on the ordinate axes for apo-form (Nδ protonation state) S2 and pNPA-bound protein for (A) Conformation 1, (B) Conformation 2 and (C) Conformation 3, Figure S6. Hydrogen bond network for water molecules explicitly considered in the QM/MM model for the pNPA-bound protein are represented as black lines for (A) Conformation 1, (B) Conformation 2. Three water molecules are indicated as red balls. The substrate is shown in green sticks, meanwhile, Cys, His and Glu are indicates as yellow, blue and red sticks, respectively, Figure S7: PES for the nucleophilic attack of the neutral thiol form of Cys-SH and deprotonated one Cys-S(^−−^) to the ester carbonyl for Conformation 1 and Conformation 2; Figure S8. Optimized geometries of TS species for (A) conformation 1, (B) conformation 2, intercepted along the PES of the hydrolytic process. For clarity, only the amino acid residues taking part in the reaction are shown, in which Cys18-His22-Glu25 residues and pNPA are represented in ball and sticks, Figure S9. Optimized geometries of TS1 species for (A) conformation 1, (B) conformation 2, intercepted along the PES of the hydrolytic process. For clarity, only the amino acid residues taking part in the reaction are shown, in which Cys18-His22-Glu25 residues and pNPA are represented in ball and sticks, Figure S10. Optimized geometries of I2A species for (A) conformation 1, (B) conformation 2, intercepted along the PES of the hydrolytic process. For clarity, only the amino acid residues taking part in the reaction are shown, in which Cys18-His22-Glu25 residues and pNPA are represented in ball and sticks, Figure S11. Free Energy Surfaces for proposed hydrolysis catalyzed by de novo protein obtained at B3LYP-D3/6-311+(2d,2p):ff14SB//B3LYP/6-31+G(d,p):ff14SB level of theory, according to the B mechanism for the two adopted conformation, Figure S12. Optimized geometries of TS3B species for (A) conformation 1, (B) conformation 2, intercepted along the PES of the hydrolytic process. For clarity, only the amino acid residues taking part in the reaction are shown, in which Cys18-His22-Glu25 residues and pNPA are represented in ball and sticks, Table S1. Calculated pKa for ionizable residues of CC-Hept-CHE. Residues fully protonated (positively charged) or deprotonated (negatively charged) are highlighted in blue and red, S3 respectively. Residues belonging to the catalytic triad are underlined, Table S2. Best pNPA docked pose for each representative structure and adopted geometrical parameters in order to predict binding mode into de novo protein, Table S3. Calculated parameters for pNPA, Table S4: Adopted geometrical filters (Å) and population (%) on each representative structure of pNPA-bound protein for the two analyzed conformations in order to select the starting point for the QM/MM investigation, Table S5: NBO charges (|e|) of selected atoms in all the species found on the PES, Table S6: Energy contributions, calculated at ONIOM[B3LYP-D3/6-311+G(2d,2p):ff14SB] level of theory, extracted to obtain Free Energy Surfaces, for each stationary point. Imaginary frequencies calculated for transition states are also reported (cm^−1^).

## Abbreviations

B3LYP	Becke-Lee-Yang-Parr
CC-Hept	Coiled Coil-Heptamer
CC-Hept-Cys-His-Glu	Coiled Coil-Heptamer-Cysteine-Histidine-Glutamate
CpHMD	Constant pH Molecular Dynamics
DFT	Density Functional Theory
FES	Free Energy Surfaces
GAFF	General Amber Force Field
MD	Molecular Dynamics
ONIOM	Our own N-layered Integrated MO and MM
pNPA	Para-NitroPhenylAcetate
QMMM	Quantum Mechanics/Molecular Mechanics
RESP	Restrained ElectroStatic Potential
RMSD	Root Mean Square Deviation
ZPE	Zero Point Energy
