# Supplementary material for: On the Catalytic Activity of the Engineered Coiled-Coil Heptamer Mimicking the Hydrolase Enzymes: Insights from a Computational Study
Source: Int J Mol Sci. 2020 Jun 26;21(12):4551. doi: 10.3390/ijms21124551 (PMC7352413; doi:10.3390/ijms21124551)
Supplement: Supplementary file 1 [file ijms-21-04551-s001.pdf]

On the catalytic activity of the engineered coiled-coil heptamer mimicking the hydrolase enzymes: insights from a computational study

Mario Prejanò, Isabella Romeo, Nino Russo*, Tiziana Marino*

Dipartimento di Chimica e Tecnologie Chimiche, Università della Calabria, 87036 Rende, CS, Italy.

correspondence should be addressed to: tiziana.marino65@unical.it; nrusso@unical.it

SUPPORTING INFORMATION

Table of contents:

Fig.S1 The initial structure adopted in the present study (PDB: 5ECZ)…...……………………………………………………………………………….S4

Fig. S2 Superposition between 10 representative structures generated by cpptraj, after 200 ns of the apo-form protein MDs with Nδ protonation state in A) top and B) front view. Each structure is represented in ribbon form. ………………………………..…..………………………………...S4

Fig. S3 Representation of the unnatural substrate pNPA binding mode into the channel of the de novo protein for each obtained representative structure. 10 poses are generated by using AutoDock Vina. The protein and the relative pNPA binding poses are shown in light grey cartoon and coloured carbon sticks, respectively. ………………..…………………………………………..…S5

Fig. S4 The pair correlation function for the sulphur of cysteine and the carbon of pNPA…………..……..…………………..………..……………..………………………..S 6

Fig. S5 Plot of the distance calculated between the centroids of all the seven Gly_1, Cys_{18} and Arg_{30} residues of the protein and the corresponding Cα of Gly, Cys and Arg of each chain as a measure of the diameter of the channel at bottom (blue line), middle and top (green and red lines), respectively, relative to the initial structure. The time of simulations (ns) and the values of the Cα distance (Å) are reported, respectively, on the abscissa and on the ordinate axes for apo-form (Nδ protonation state) and *p*NPA-bound protein for **A**) Conformation 1, **B**) Conformation 2 and **C**) Conformation 3……………………………...............…………………….……………………S7

Fig. S6 Hydrogen bond network for water molecules explicitly considered in the QM/MM model for the pNPA-bound protein are represented as black lines for A) Conformation 1, B) Conformation 2. Three water molecules are indicated as red balls. The substrate is shown in green sticks, meanwhile, Cys, His and Glu are indicates as yellow, blue and red sticks, respectively……………………………………………………………...……....S 7

Fig. S7 PES for the nucleophilic attack of the neutral thiol form of Cys-SH and deprotonated one Cys-S(-) to the ester carbonyl for Conformation 1 and Conformation 2….. …..…………...…...S8

Fig. S8 Optimized geometries of **TS** species for A) conformation 1, B) conformation 2, intercepted along the PES of the hydrolytic process. For clarity, only the amino acid residues taking part in the reaction are shown, in which Cys18-His22-Glu25 residues and pNPA are represented in ball and sticks……………………..………………………………………………..…..….S 8

Fig. S9 Optimized geometries of **TS1** species for A) conformation 1, B) conformation 2, intercepted along the PES of the hydrolytic process. For clarity, only the amino acid residues taking part in the reaction are shown, in which Cys18-His22-Glu25 residues and pNPA are represented in ball and sticks……………………………………………….…..S9

Fig. S10 Optimized geometries of **I2A** species for A) conformation 1, B) conformation 2, intercepted along the PES of the hydrolytic process. For clarity, only the amino acid residues taking part in the reaction are shown, in which Cys18-His22-Glu25 residues and pNPA are represented in ball and sticks.. ……………………………………………………………………………...…..S9

Fig. S11 Free Energy Surfaces for proposed hydrolysis catalyzed by de novo protein obtained at B3LYP-D3/6-311+(2d,2p):ff14SB//B3LYP/6-31+G(d,p):ff14SB level of theory, according to the B mechanism for the two adopted conformation……………………..………...…………….…..S10

Fig. S12 Optimized geometries of **TS3B** species for A) conformation 1, B) conformation 2, intercepted along the PES of the hydrolytic process. For clarity, only the amino acid residues taking part in the reaction are shown, in which Cys18-His22-Glu25 residues and pNPA are represented in ball and sticks...……………………..……………………………………………………..S10

Table S1. Calculated pKa for ionizable residues of CC-Hept-CHE. Residues fully protonated (positively charged) or deprotonated (negatively charged) are highlighted in blue and red, respectively. Residues belonging to the catalytic triad are underlined………………………………………………………………..…..………..…....S 11

Table S2. Best pNPA docked pose for each representative structure and adopted geometrical parameters in order to predict binding mode into de novo

protein……………………………………………………………………………….……......S1
2

Table S3. Calculated parameters for pNPA…...S13

Table S4. Adopted geometrical filters on each representative structure of pNPA-bound protein for the two analyzed conformations in order to select the starting point for the QM/MM
investigation…………………………...S1
4

Table S5. NBO charges (|e|) of selected atoms in all the species found on the PES……………………………..…...S15

Table S6. Energy contributions, calculated at ONIOM[B3LYP-D3/6-311+G(2d,2p):ff14SB] level of theory, extracted to obtain Free Energy Surfaces, for each stationary point. Imaginary frequencies calculated for transition states are also reported (cm^{-1})…………….
………………………….…S15

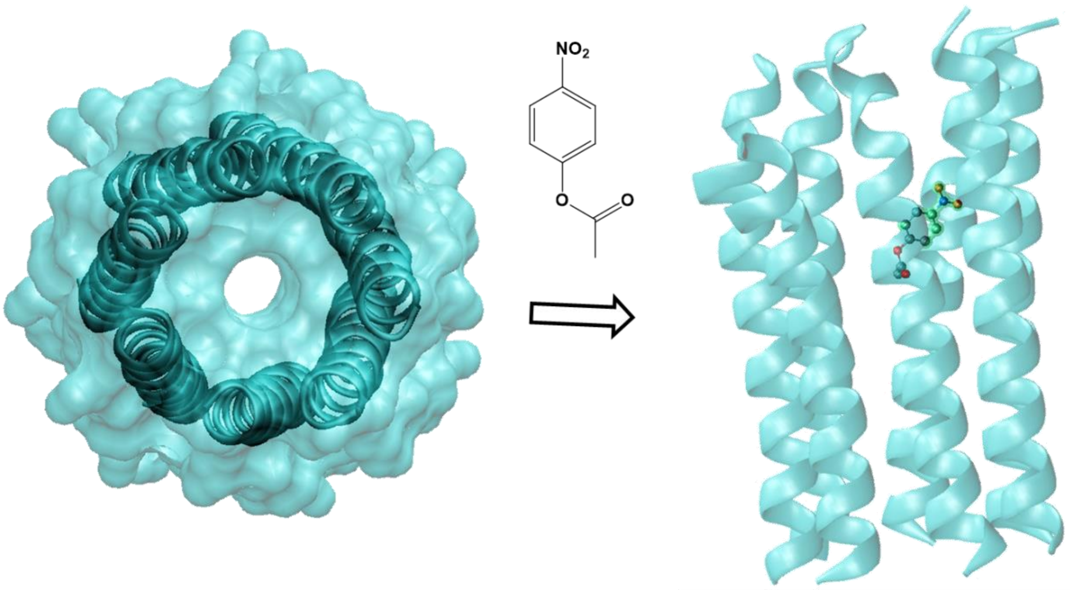

Figure S1. The initial structure adopted in the present study (PDB: 5ECZ).

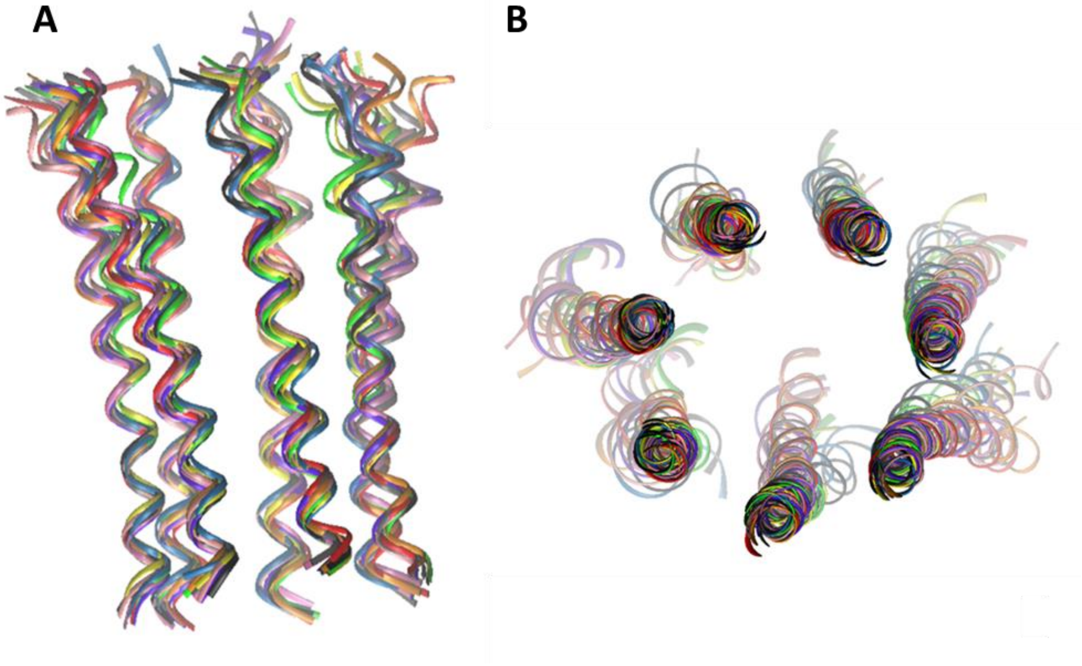

Fig. S2 Superposition between 10 representative structures generated by *cpptraj*, after 200 ns of the apo-form protein MDs with Nδ protonation state in **A**) top and **B**) front view. Each structure is represented in ribbon form.

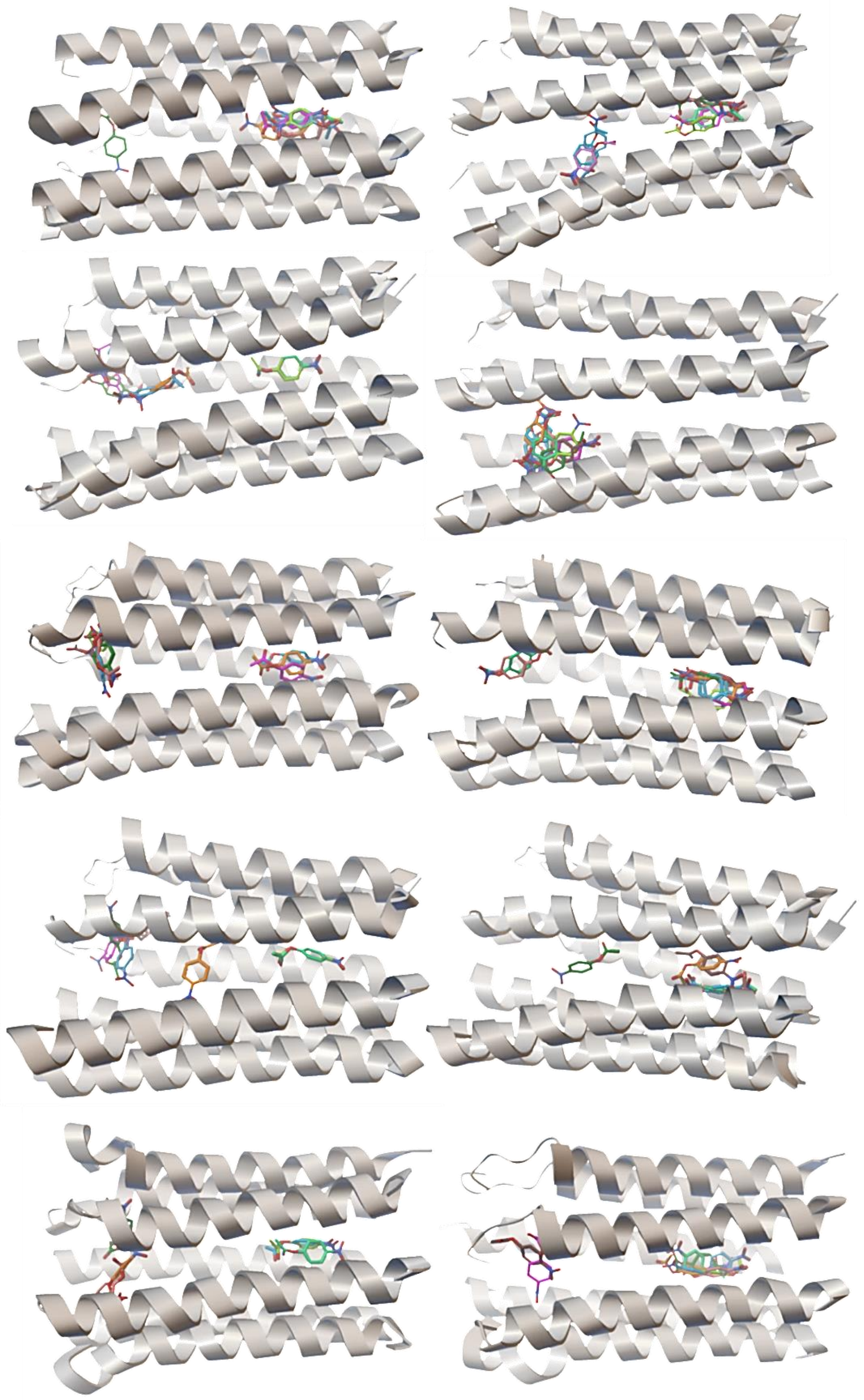

Fig. S3 Representation of the unnatural substrate *p*NPA binding mode into the channel of the *de novo* protein for each obtained representative structure. 10 poses are generated by using AutoDock Vina. The protein and the relative *p*NPA binding poses are shown in light grey cartoon and coloured carbon sticks, respectively.

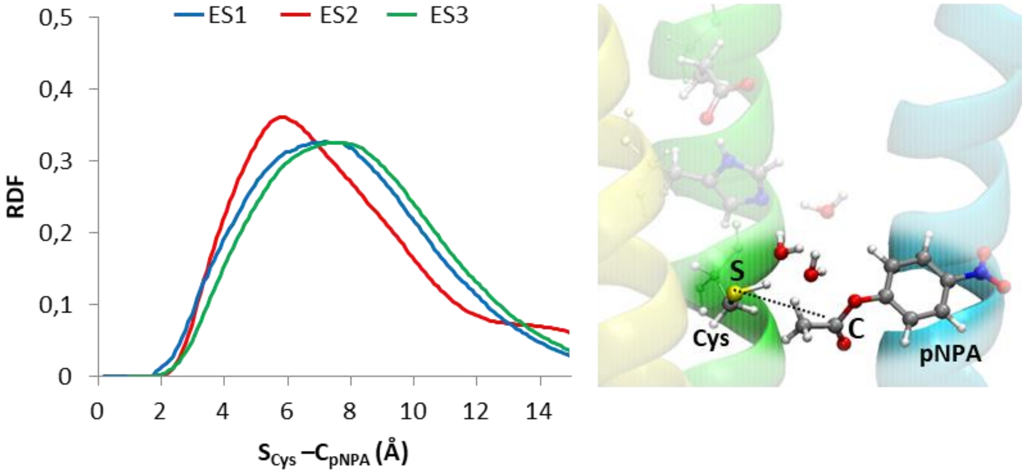

Fig. S4. The pair correlation function for the sulphur of cysteine and the carbon of pNPA.

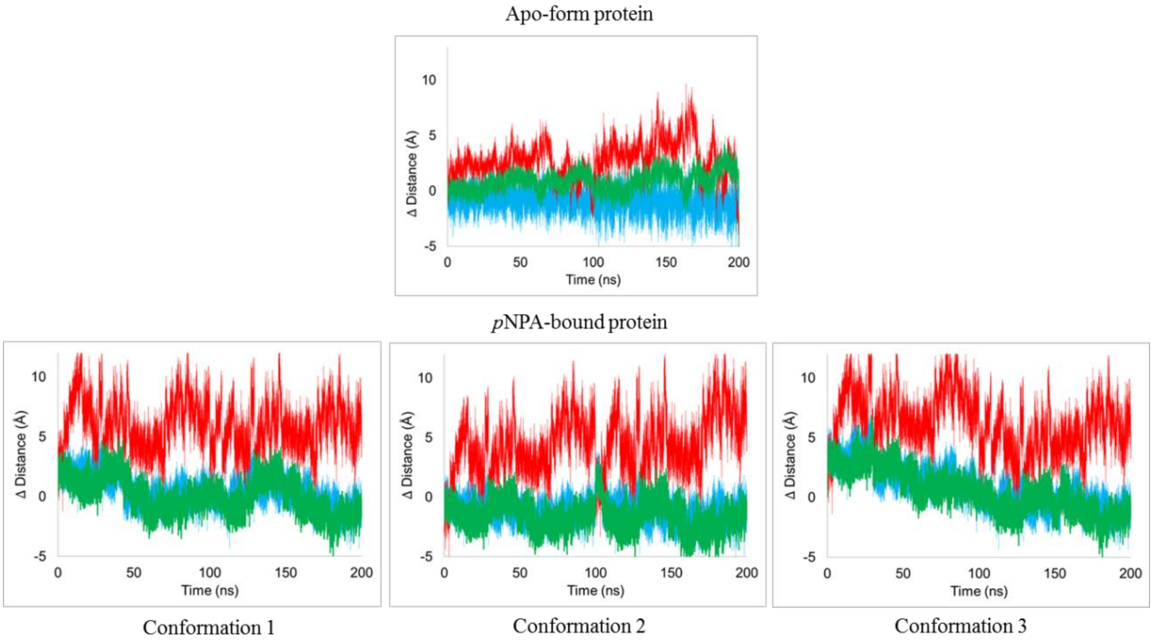

Fig. S5 Plot of the distance calculated between the centroids of all the seven Gly_1, Cys_{18} and Arg_{30} residues of the protein and the corresponding Cα of Gly, Cys and Arg of each chain as a measure of the diameter of the channel at bottom (blue line), middle and top (green and red lines), respectively, relative to the initial structure. The time of simulations (ns) and the values of the Cα distance (Å) are reported, respectively, on the abscissa and on the ordinate axes for apo-form (Nδ protonation state) and *p*NPA-bound protein for **A**) Conformation 1, **B**) Conformation 2 and **C**) Conformation 3.

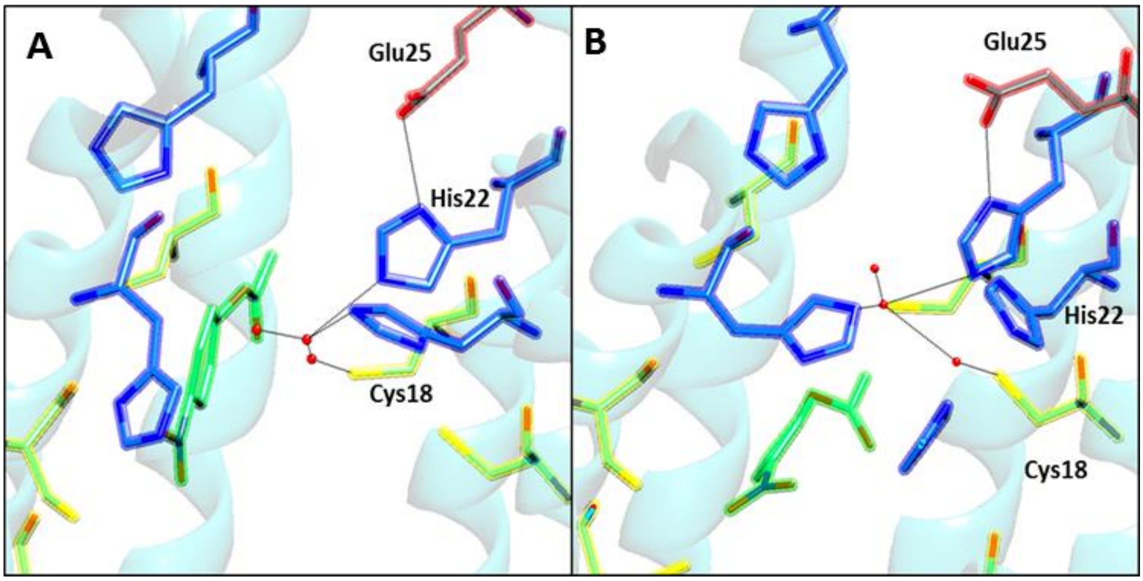

Fig. S6. Hydrogen bond network for water molecules explicitly considered in the QM/MM model for the pNPA-bound protein are represented as black lines for A) Conformation 1, B) Conformation 2. Three water molecules are indicated as red balls. The substrate is shown in green sticks, meanwhile, Cys, His and Glu are indicates as yellow, blue and red sticks, respectively.

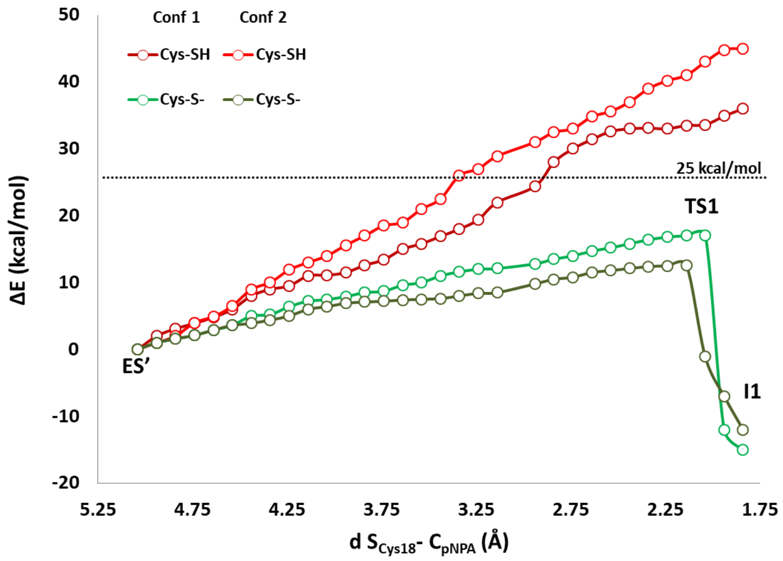

Fig. S7 PES for the nucleophilic attack of the neutral thiol form of Cys-SH and deprotonated one Cys-S$^{(-)}$ to the ester carbonyl for **A**) Conformation 1, **B**) Conformation 2.

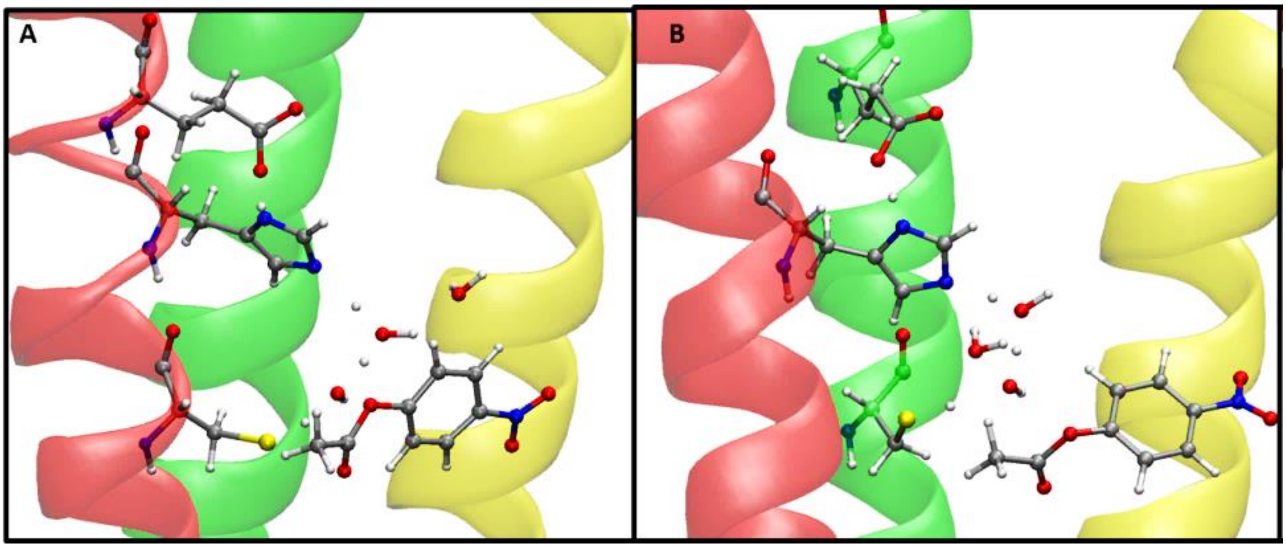

Fig. S8 Optimized geometries of **TS** species for **A**) conformation 1, **B**) conformation 2, intercepted along the PES of the hydrolytic process. For clarity, only the amino acid residues taking part in the reaction are shown, in which Cys_{18}-His_{22}-Glu_{25} residues and *p*NPA are represented in ball and sticks.

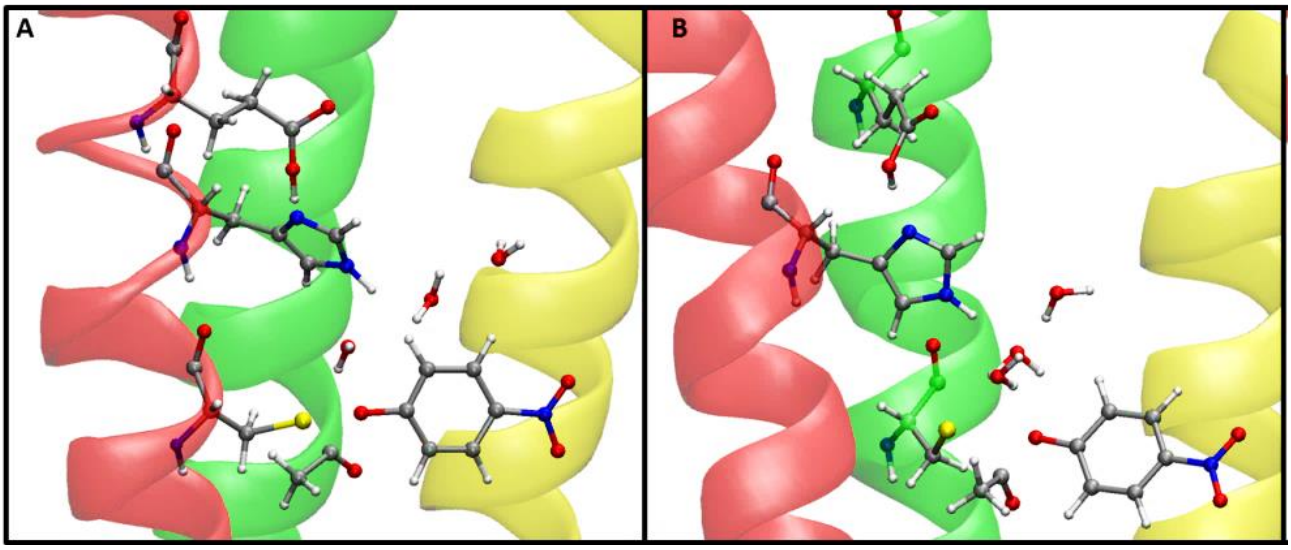

Fig. S9. Optimized geometries of **TS1** species for **A**) conformation 1, **B**) conformation 2, intercepted along the PES of the hydrolytic process. For clarity, only the amino acid residues taking part in the reaction are shown, in which Cys_{18}-His_{22}-Glu_{25} residues and *p*NPA are represented in ball and sticks.

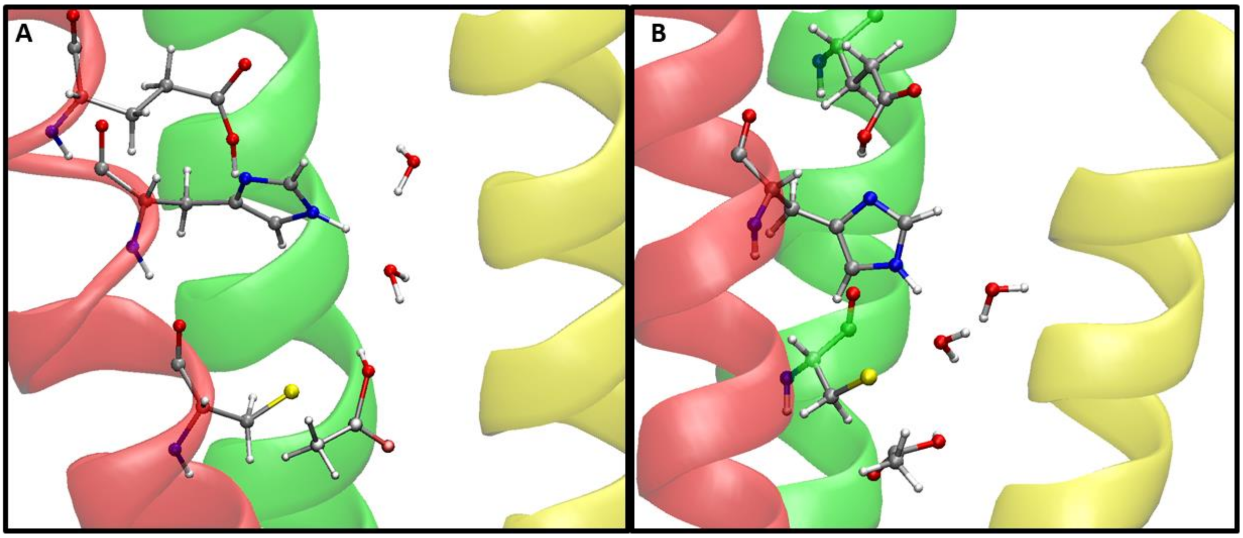

Fig. S10. Optimized geometries of **I2A** species for **A**) conformation 1, **B**) conformation 2, intercepted along the PES of the hydrolytic process. For clarity, only the amino acid residues taking part in the reaction are shown, in which Cys_{18}-His_{22}-Glu_{25} residues and *p*NPA are represented in ball and sticks.

In the channel B the hydrolysis of thioester involves the w1 that donates the proton to the sulfur atom (O_{w1}--H 1.61 Å and H--S 1.78 Å) producing the O_{w1}--C bond formation (1.60 Å for conformation 2) and the S--C bond cleavage (1.98 Å for conformation 2) in concerted way. The corresponding transition state (**TS2B**) has in all cases one imaginary frequency of 750i cm^{-1} and a Gibbs activation energy of 31.8 and 22.5 kcal/mol (Figure S12) for conformation 1 and 2 . The **I2B** resulting species are almost isoenergetic with **I1** for the conformation 1 and 2. The next step accounts for restoring of the catalytic center through a concerted proton cascade involving w2, His, Glu and pNPA (see **TS3B** in Figure S13). The imaginary frequencies of about 1000i cm^{-1} for the two paths confirm the main role played by water in this rearrangement. The final reaction Gibbs energy results to be exothermic for configuration 1 and 2 (Figure S12).

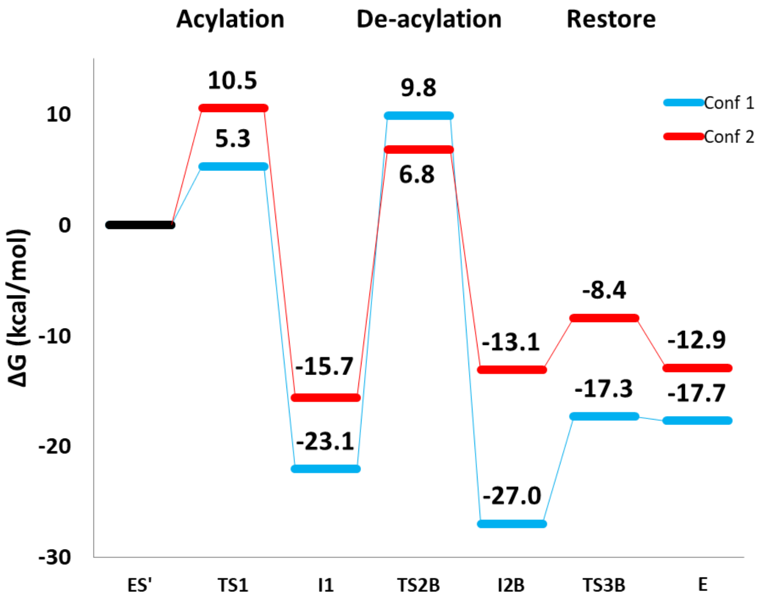

Fig. S11 Free Energy Surfaces for proposed hydrolysis catalyzed by de novo protein obtained at B3LYP-D3/6-311+(2d,2p):ff14SB//B3LYP/6-31+G(d,p):ff14SB level of theory, according to the B mechanism for the two adopted conformations.

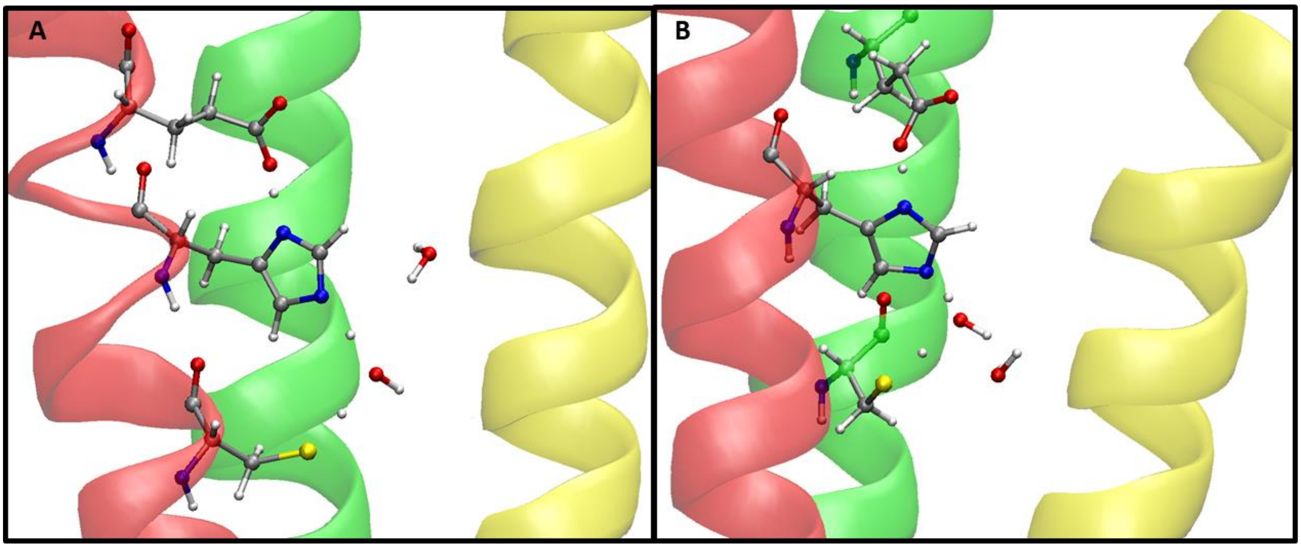

Fig. S12 Optimized geometries of **TS3B** species for **A**) conformation 1, **B**) conformation 2, intercepted along the PES of the hydrolytic process. For clarity, only the amino acid residues taking part in the reaction are shown, in which Cys_{18}-His_{22}-Glu_{25} residues and *p*NPA are represented in ball and sticks.

Table S1. Calculated pKa f or ionizable residues of CC-Hept-CHE. Residues fully protonated (positively charged) or deprotonated (negatively charged) are highlighted in blue and red, respectively. Residues belonging to the catalytic triad are underlined.

	A	B	C	D	E	F	G
Glu3	1.5	1.3	1.5	1.3	1.4	1.2	1.8
Lys6	10.0	10.1	9.9	8.8	10.1	10.0	10.1
Arg9	≈12.0	≈12.0	≈12.0	≈12.0	≈12.0	≈12.0	≈12.0
Glu10	1.9	0.8	0.5	1.3	2.3	0.5	2.1
Lys13	10.1	10.1	10.1	10.1	10.0	10.0	10.0
Arg16	≈12.0	≈12.0	≈12.0	≈12.0	≈12.0	≈12.0	12.0

Glu17	1.6	2.1	2.1	2.2	1.7	2.0	1.5
**Cys18***	≈12.0	≈12.0	≈12.0	≈12.0	≈12.0	≈12.0	≈12.0
**His22***	5.9	4.4	5.5	6.1	5.5	5.4	4.4
Arg23	≈12.0	≈12.0	≈12.0	≈12.0	≈12.0	≈12.0	≈12.0
Glu24	6.4	7.0	8.3	6.8	5.7	5.4	8.0
**Glu25***	3.4	2.8	3.0	0.2	3.8	2.9	3.1
Lys27	10.9	10.8	10.7	11.5	11.0	11.2	10.8
Arg30	≈12.0	≈12.0	≈12.0	≈12.0	≈12.0	≈12.0	≈12.0

Table S2. Best *p*NPA docked pose for each representative structure and adopted geometrical parameters in order to predict binding mode into *de novo* protein.

	Vina binding scores (kcal/mol)	S_{Cys} - C_{pNPA} atomic distance (Å)	S_{Cys} – $N_{\varepsilon His}$ atomic distance (Å)	$N_{\delta His}$ – O_{Glu} Atomic distance (Å)
Cluster 1	-5.4	13.697	3.472	8.584
Cluster 2	-5.5	4.355	3.795	2.815
Cluster 3	-5.7	13.487	5.732	12.688
Cluster 4	-6.3	5.796	6.306	2.855
Cluster 5	-5.8	11.351	3.357	9.517
Cluster 6	-5.0	11.200	6.098	6.066
Cluster 7	-5.3	13.027	5.273	11.417
Cluster 8	-5.3	4.566	5.356	9.883
Cluster 9	-5.2	11.066	3.553	8.030
Cluster 10	-4.9	7.732	7.963	5.774

Table S3. Calculated parameters for *p*NPA.

Atomtype	Charge
Ca	-0.010
Ha	0.193
No	0.813
O	-0.473
Os	-0.524
C	0.938
c3	-0.558
Hc	0.162

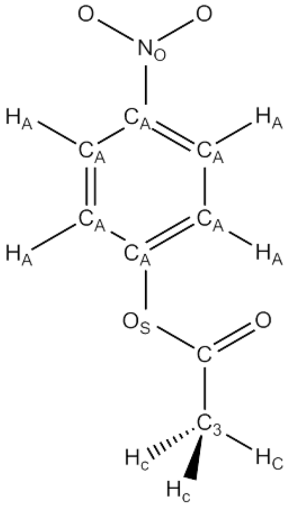

Bond	K_i / kcal mol^{-1} Å^{-2}	l_0/Å
ca-ca	461.10	1.398
ca-no	321.70	1.469
ca-ha	345.80	1.086
no-o	741.80	1.226
ca-os	376.60	1.370
os-c	390.80	1.358
c -c3	313.00	1.524
c –o	637.70	1.218
c3-hc	330.60	1.097

Angle	K_{an}/ Kcal mol^{-1} rad^{-2}	θ_0/deg
ca-ca-ca	66.620	120.020
ca-ca-ha	48.180	119.880
ca-no-o	68.700	117.760
ca-ca-no	66.770	119.010
ca-ca-os	69.580	119.200
o -no-o	76.730	125.080
ca-os-c	63.410	121.150
os-c -c3	68.890	110.720
os-c -o	75.320	123.250
c -c3-hc	46.930	108.770
c3-c -o	67.400	123.200
hc-c3-hc	39.400	107.580

Dihedral	d	V_n/ Kcal mol^{-1} rad^{-2}	γ/deg	n
ca-ca-ca-ha	1	3.625	180.000	2.000
ca-ca-ca-ca	1	3.625	180.000	2.000
ca-ca-no-o	1	0.600	180.000	2.000
ca-ca-ca-os	1	3.625	180.000	2.000
ca-ca-ca-no	1	3.625	180.000	2.000
ca-ca-os-c	1	0.900	180.000	2.000
ha-ca-ca-ha	1	3.625	180.000	2.000
ha-ca-ca-os	1	3.625	180.000	2.000
ha-ca-ca-no	1	3.625	180.000	2.000
ca-os-c -c3	1	2.700	180.000	2.000
ca-os-c -o	1	2.700	180.000	2.000
os-c -c3-hc	1	0.000	180.000	2.000
hc-c3-c -o	1	0.800	0.000	-1.000
hc-c3-c -o	1	0.000	0.000	-2.000

hc-c3-c -o	1	0.080	180.000	3.000

Improper	V_n/ Kcal mol^{-1} rad^{-2}	γ/deg	n
ca-ca-ca-no	1.1	180.0	2.0
ca-ca-ca-ha	1.1	180.0	2.0
ca-o -no-o	1.1	180.0	2.0
ca-ca-ca-os	1.1	180.0	2.0
c3-o -c -os	10.5	180.0	2.0

VdW	r_0/Å	ε/ kcal mol^{-1}
ca	1.908	0.0860
ha	1.459	0.0150
no	1.824	0.1700
o	1.6612	0.2100
os	1.6837	0.1700
c	1.908	0.0860
c3	1.908	0.1094
hc	1.487	0.0157

Table S4. Adopted geometrical filters on each representative structure of *p*NPA-bound protein for the two analyzed conformations in order to select the starting point for the QM/MM investigation.

	Conformation 1			Conformation 2			Conformation 3		
	S_{Cys} - C_{pNPA} atomic distance (Å)	S_{Cys} – $N_{\varepsilon His}$ atomic distance (Å)	$N_{\delta His}$ – O_{Glu} atomic distance (Å)	S_{Cys} - C_{pNPA} atomic distance (Å)	S_{Cys} – $N_{\varepsilon His}$ atomic distance (Å)	$N_{\delta His}$ – O_{Glu} atomic distance (Å)	S_{Cys} - C_{pNPA} atomic distance (Å)	S_{Cys} – $N_{\varepsilon His}$ atomic distance (Å)	$N_{\delta His}$ – O_{Glu} atomic distance (Å)
**Cluster 1**	6.44	9.80	8.11	4.08	7.24	4.697	3.98	6.68	5.78
**Cluster 2**	**5.46**	**5.66**	**2.79**	4.28	7.48	4.73	3.54	5.32	10.62
**Cluster 3**	3.70	6.57	7.78	5.02	7.57	5.05	4.48	5.29	14.58
**Cluster 4**	7.09	3.82	6.66	**5.77**	**5.35**	**2.89**	10.43	4.65	11.46
**Cluster 5**	4.69	5.76	8.13	5.71	5.59	6.80	4.89	8.64	11.61
**Cluster 6**	4.89	7.65	3.13	5.29	8.18	4.87	5.54	4.28	2.71
**Cluster 7**	7.28	6.82	5.70	4.59	6.98	4.90	6.93	7.72	4.70
**Cluster 8**	3.99	6.36	7.74	4.09	7.58	4.91	6.35	5.01	15.46
**Cluster 9**	5.71	4.62	8.24	5.84	3.69	9.69	4.98	4.31	6.63

**Cluster 10**	6.12	5.66	6.53	5.64	7.23	6.68	5.07	5.12	6.67

Table S5. NBO charges (|e|) of selected atoms in all the species found on the PES.

	ES	TS1	I1	TS2	I2
S_{Cys}	-0,138	-0,166	-0,652	-0,239	0,174
C_{NPA}	0,837	0,841	0,820	0,616	0,401
O_{w1}	-1,013	-1,101	-1,090	-1,066	-1,041
$O1_{NPA}$	-0,593	-0,599	-0,603	-0,667	-0,577
$O2_{NPA}$	-0,559	-0,563	-0,535	-0,604	-0,809
	TS3A	I3A	TS4A	EP	
S_{Cys}	-0,138	-0,166	-0,652	-0,239	
C_{NPA}	0,837	0,841	0,820	0,616	
O_{w1}	-1,013	-1,101	-1,090	-1,066	
$O1_{NPA}$	-0,593	-0,599	-0,603	-0,667	
$O2_{NPA}$	-0,559	-0,563	-0,535	-0,604	
	TS3B	I3B	TS4B	I4B	TS5B
S_{Cys}	0,080	-0,261	-0,501	-0,715	-0,273
C_{NPA}	0,402	0,584	0,699	0,819	0,820
O_{w1}	-0,838	-0,850	-0,816	-0,747	-0,769
$O1_{NPA}$	-0,732	-0,749	-0,681	-0,615	-0,693
$O2_{NPA}$	-0,842	-0,788	-0,755	-0,789	-0,748

Table S6. Energy contributions, calculated at ONIOM[B3LYP-D3/6-311+G(2d,2p):ff14SB] level of theory, extracted to obtain Free Energy Surfaces, for each stationary point. Imaginary frequencies calculated for transition states are also reported (cm^{-1}).

Species	Conformation	E_{B3LYP} (a.u.)	E_{ZPE}(a.u.)	E_{D3} (a.u.)	-TΔS (kcal/mol)	E_{total} (a.u)	ṽ (cm^{-1})
**ES**	1	-6065.269687	29.113458	-0.179546	0.0	-6036.335829	
	2	-6065.276575	29.114817	-0.177247	0.0	-6036.339006	
**TS1**	1	-6065.226492	29.103458	-0.179946	1.2	-6036.30498	800*i*
	2	-6065.250377	29.104580	-0.177202	-0.9	-6036.322998	892*i*
**I1**	1	-6065.299978	29.115458	-0.180146	-0.4	-6036.325544	

	2	-6065.266732	29.112009	-0.178182	-0.2	-6.036.33291	
TS2	1	-6065.25711	29.116487	-0.179815	1.7	-6036.320438	119*i*
	2	-6065.256521	29.115570	-0.174065	-1.0	-6036.315019	153*i*
I2	1	-6065.301561	29.118462	-0.175890	1.1	-6036.362859	
	2	-6065.304601	29.119100	-0.171312	0.1	-6036.356814	
TS3A	1	-6065.296611	29.165740	-0.177796	-1.1	-6036.308667	680*i*
	2	-6065.270381	29.117517	-0.174539	-0.2	-6036.327403	752*i*
TS3B	1	-6065.319528	29.15874	-0.173698	-0.3	-6036.334486	600*i*
	2	-6065.266646	29.112971	-0.172782	0.1	-6036.326458	559*i*
I3A	1	-6065.312226	29.115701	-0.169985	-1.6	-6036.366510	
	2	-6065.300780	29.115517	-0.170976	1.3	-6036.356239	
I3B	1	-6065.277880	29.117852	-0.168562	0.9	-6036.32859	
	2	-6065.273341	29.116018	-0.170753	0.1	-6036.328076	
TS4A	1	-6065.294370	29.109483	-0.169589	-0.5	-6036.354476	998*i*
	2	-6065.284532	29.107267	-0.168867	-0.3	-6036.346132	1002*i*
TS4B	1	-6065.254587	29.115754	-0.170123	-0.7	-6036.308956	81*i*
	2	-6065.270212	29.115973	-0.172060	0.2	-6036.326298	79*i*
I4B	1	-6065.285765	29.116895	-0.171456	0.7	-6036.340326	
	2	-6065.279843	29.116563	-0.172460	0.1	-6036.335741	

**TS5B**	1	-6065.275598	29.104528	-0.166555	0.9	-6036.337625	860*i*
	2	-6065.271466	29.106182	-0.163046	-0.3	-6036.328330	882*i*
**EP**	1	-6065.299092	29.111256	-0.167222	0.5	-6036.355058	
	2	-6065.298616	29.114800	-0.169381	-0.4	-6036.353198	
**pNP**		-512.141645	0.107308	-0.010257		-512.042105	
**CH_3COOH**		-229.172132	0.061779	-0.003000		-229.111027	
